# Supplementary figures and images for: Cyclic AMP is a critical mediator of intrinsic drug resistance and fatty acid metabolism in M. tuberculosis
Source: eLife. 2023 Feb 22;12:e81177. doi: 10.7554/eLife.81177 (PMC9995111; doi:10.7554/eLife.81177)

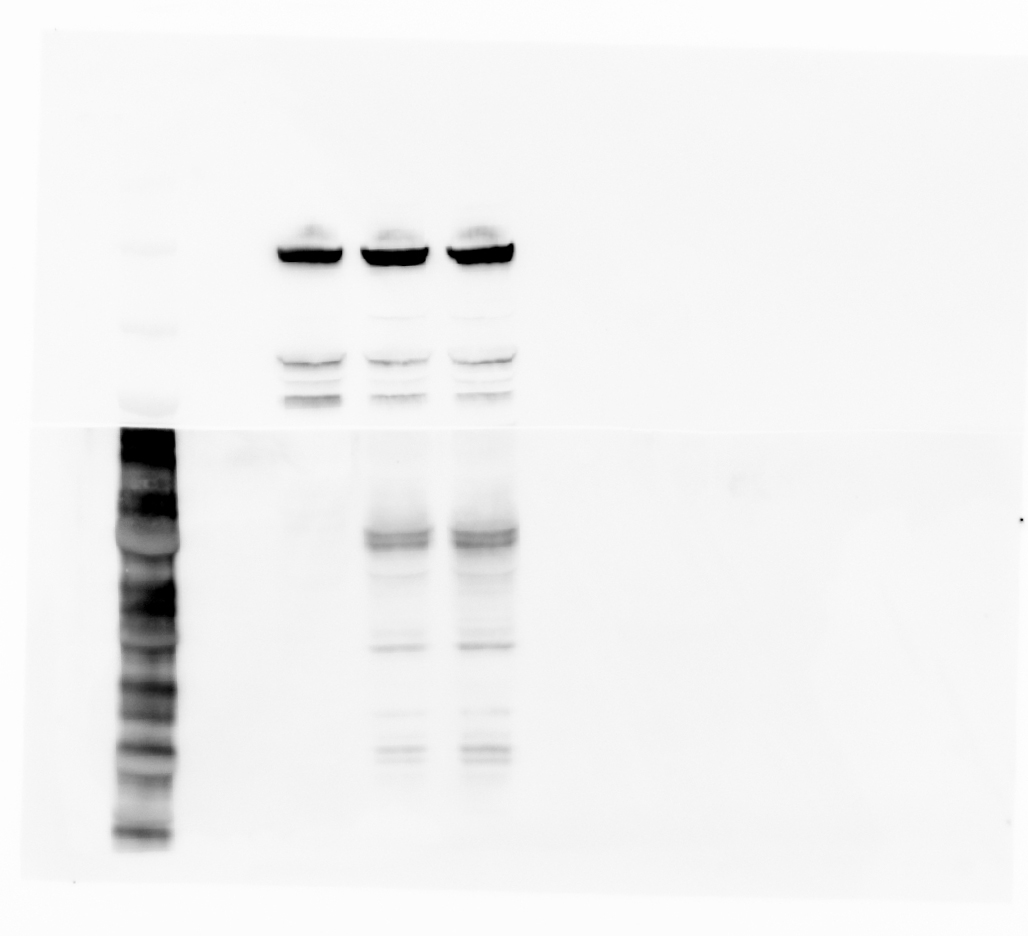

Supplement: Figure 5—figure supplement 1—source data 1. [file elife-81177-fig5-figsupp1-data1.zip › Figure 5 - supplement 1 - original.jpg]

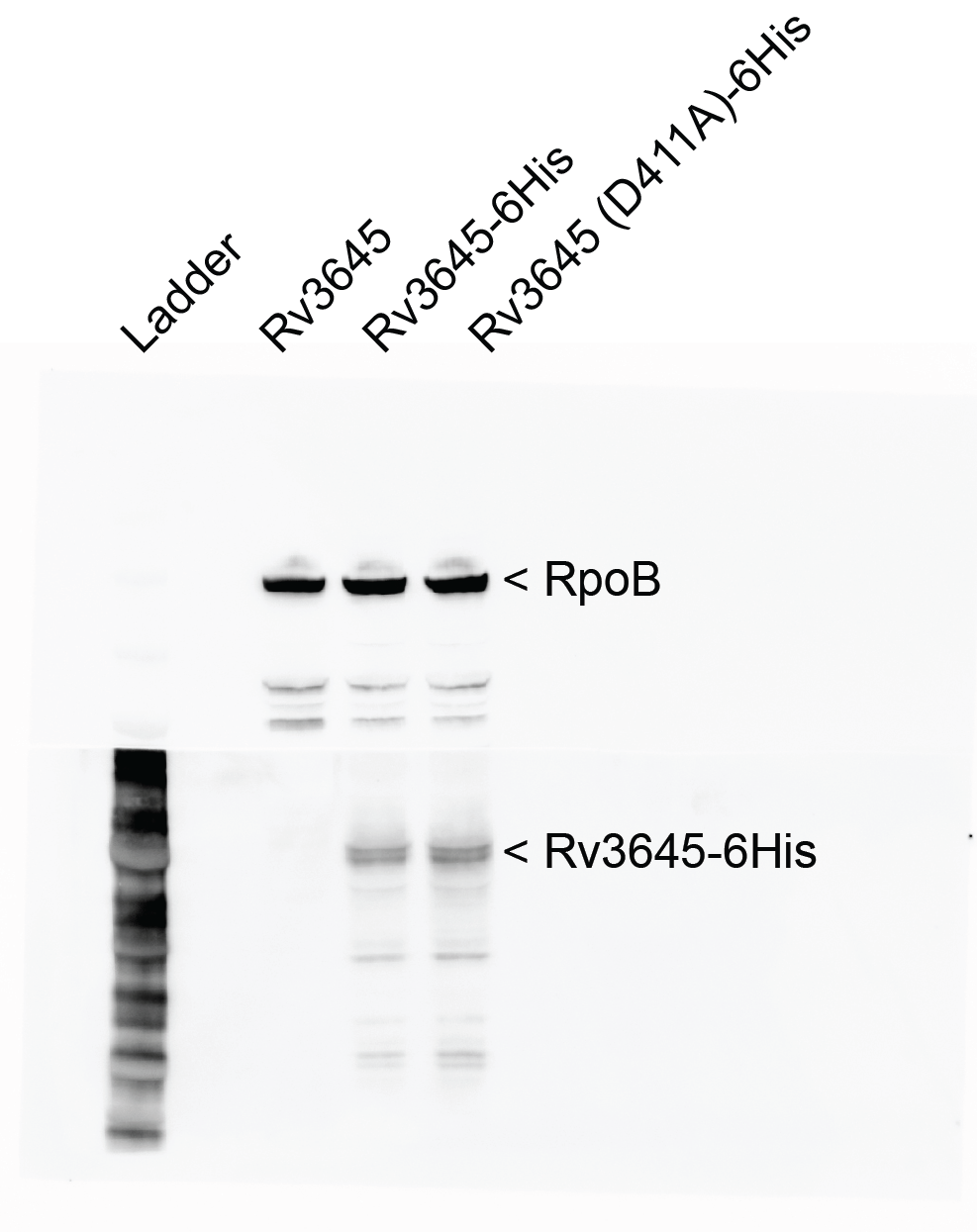

Supplement: Figure 5—figure supplement 1—source data 1. [file elife-81177-fig5-figsupp1-data1.zip › Figure 5 - supplement 1 - uncropped, labelled.png]

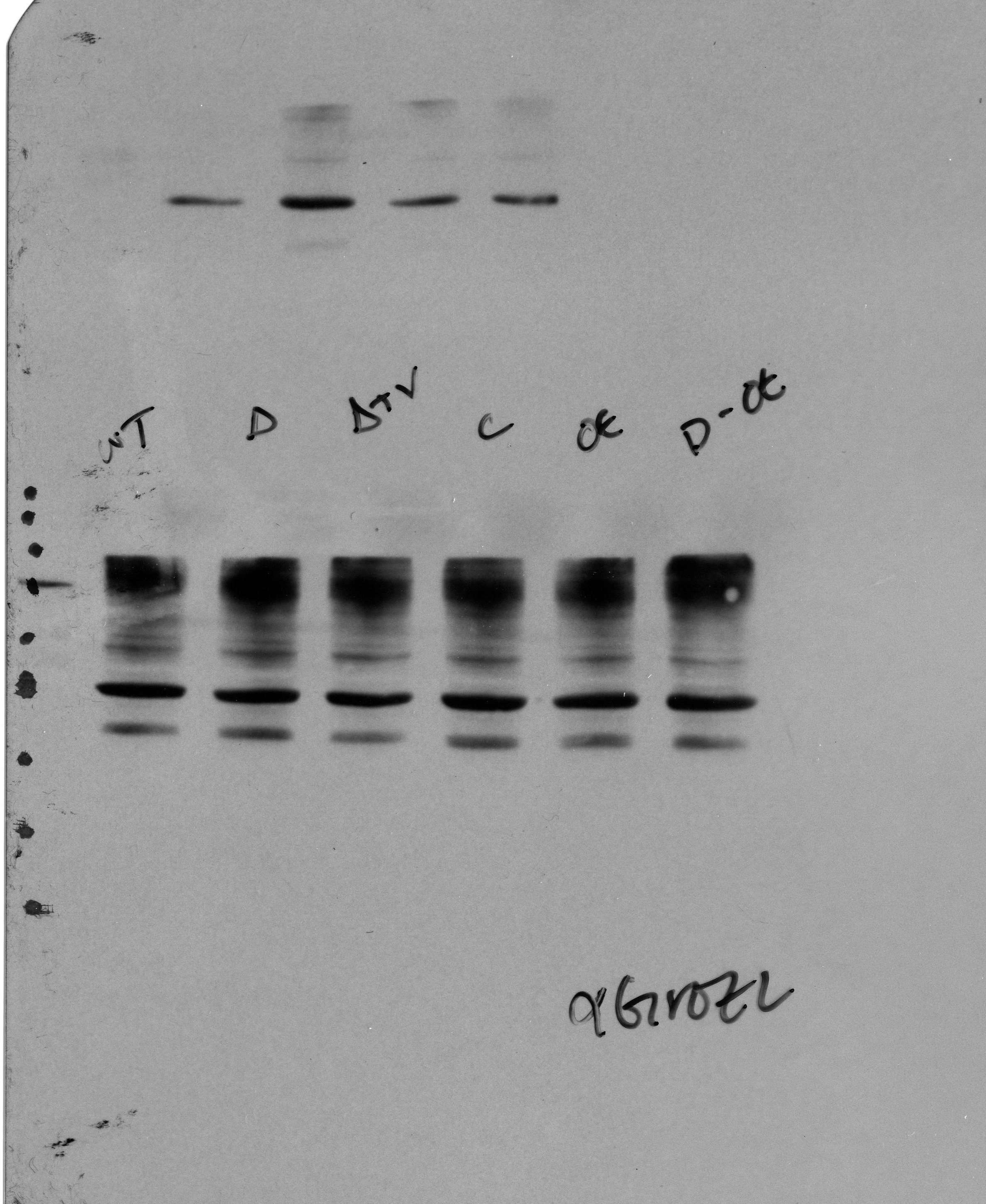

Supplement: Figure 5—figure supplement 3—source data 1. [file elife-81177-fig5-figsupp3-data1.zip › Figure 5 - figure supplement 3 - GroEL, original.jpg]

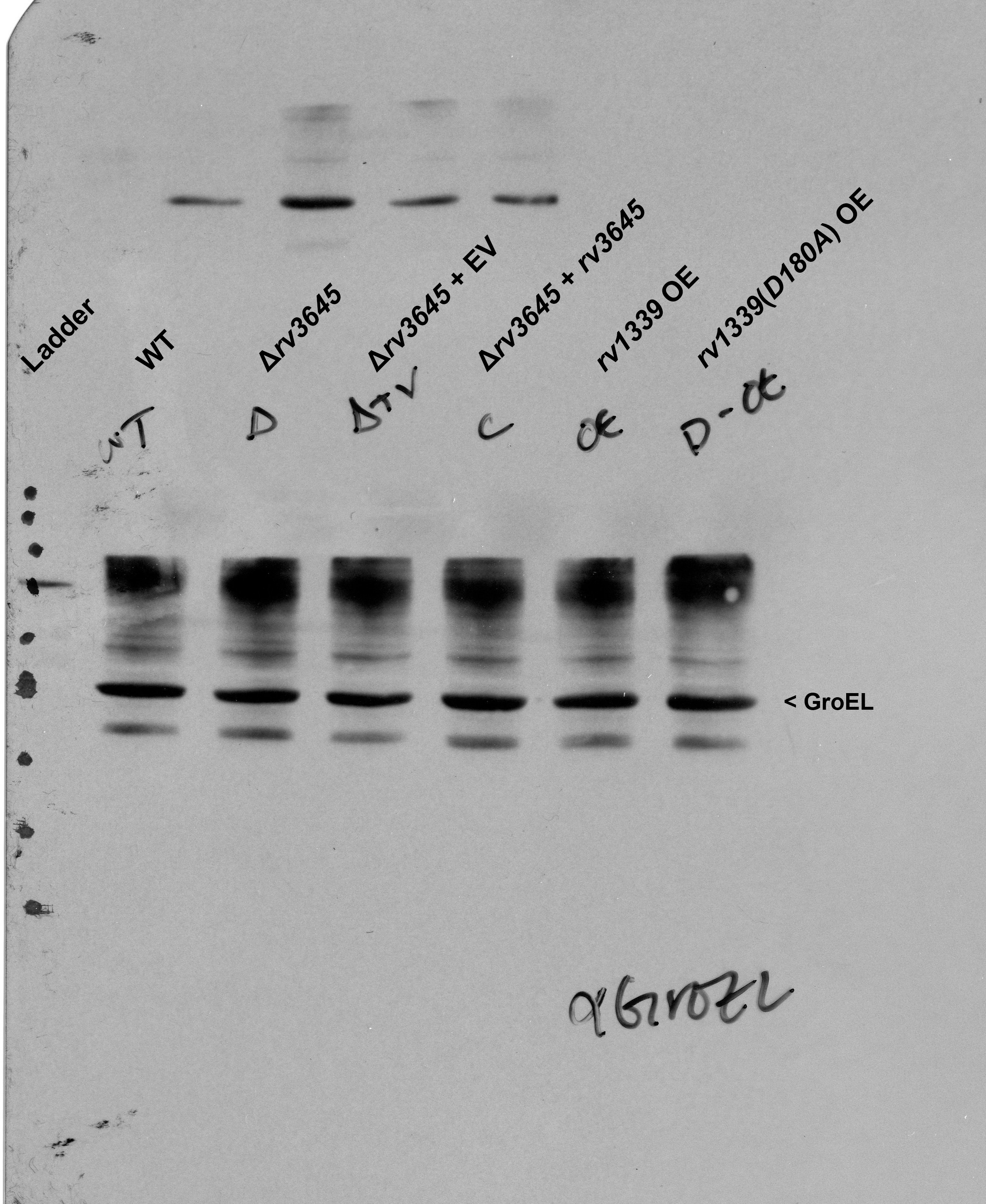

Supplement: Figure 5—figure supplement 3—source data 1. [file elife-81177-fig5-figsupp3-data1.zip › Figure 5 - figure supplement 3 - GroEL, uncropped, labelled.png]

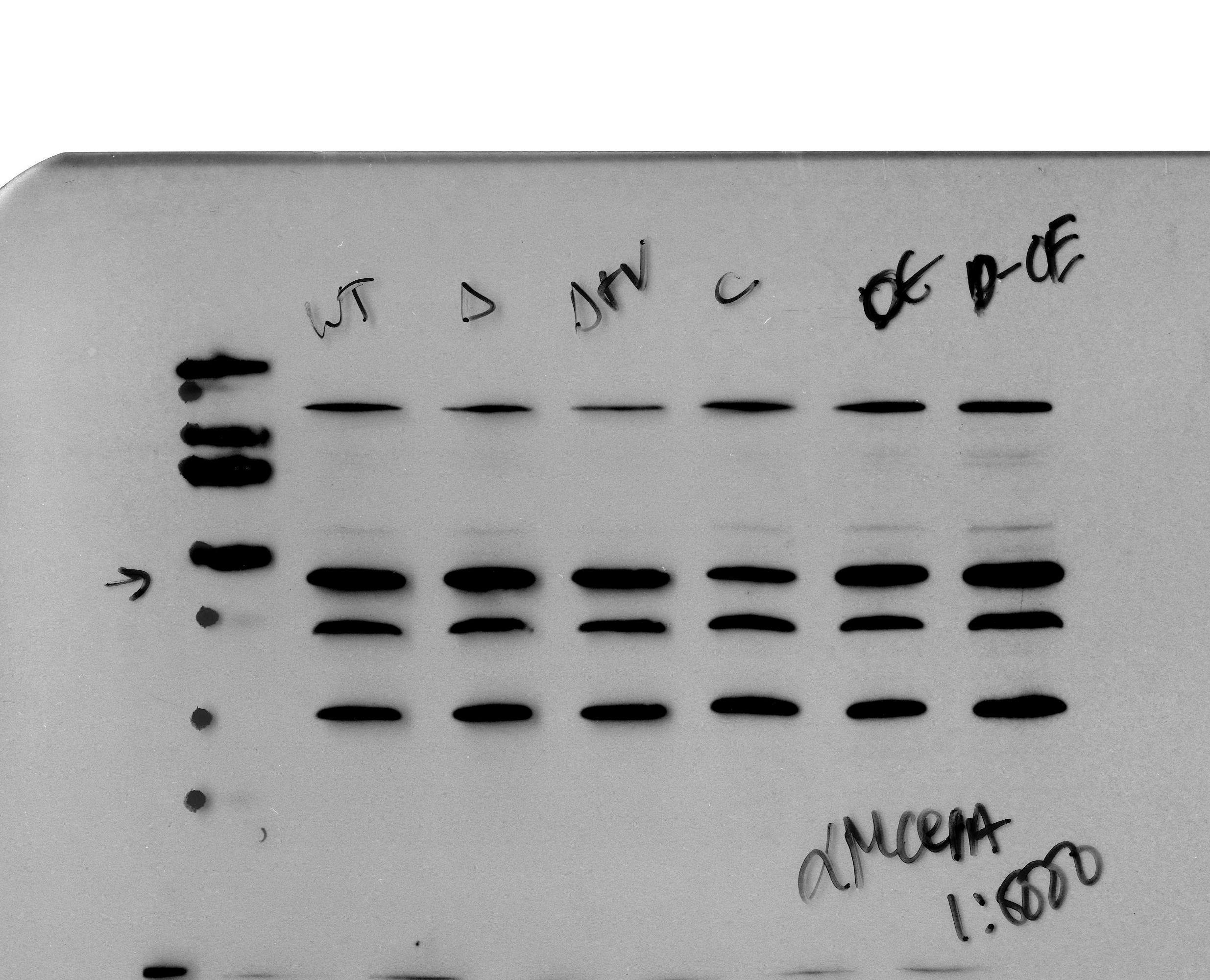

Supplement: Figure 5—figure supplement 3—source data 1. [file elife-81177-fig5-figsupp3-data1.zip › Figure 5 - figure supplement 3 - Mce1A, original.png]

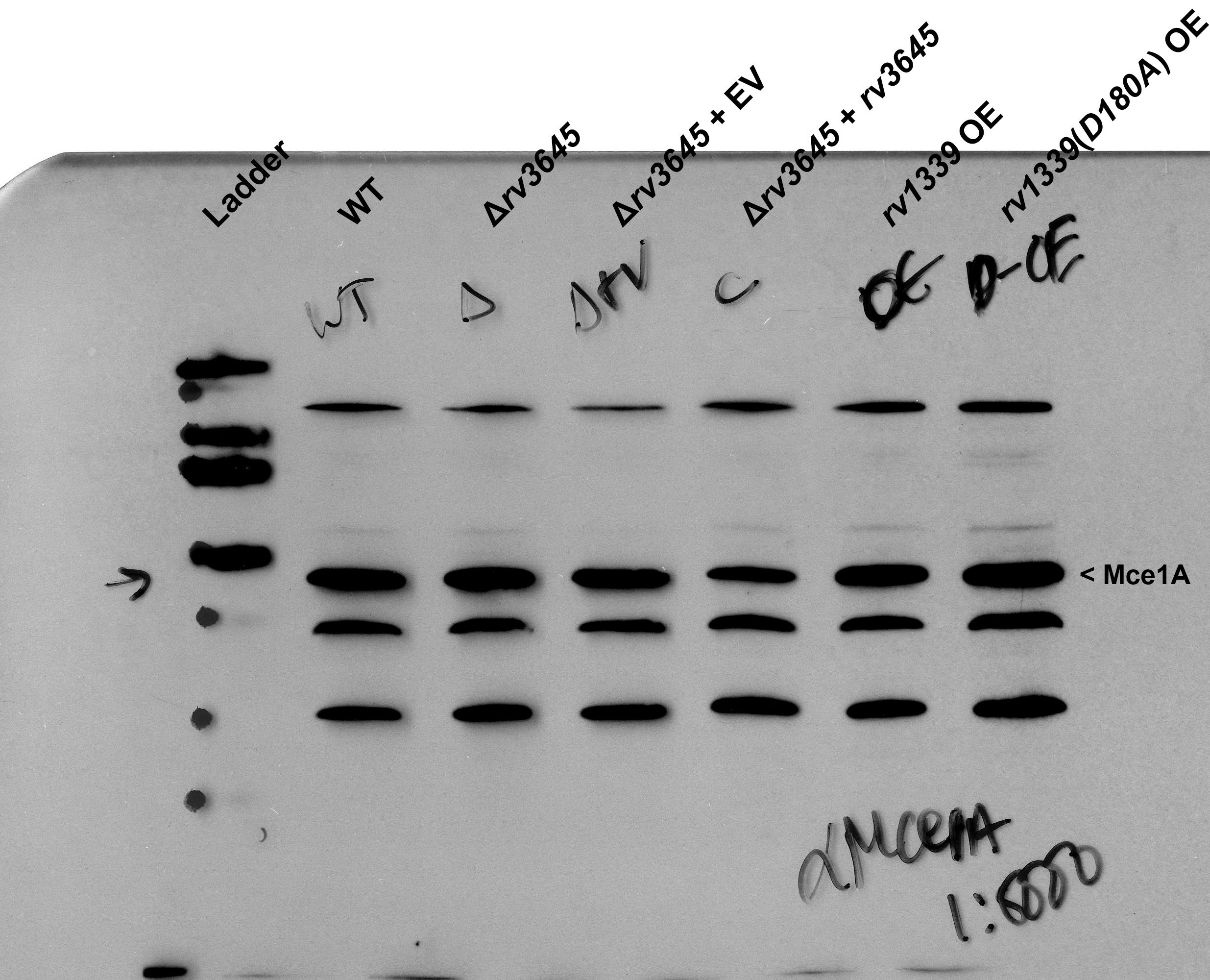

Supplement: Figure 5—figure supplement 3—source data 1. [file elife-81177-fig5-figsupp3-data1.zip › Figure 5 - figure supplement 3 - Mce1A, uncropped, labelled.png]

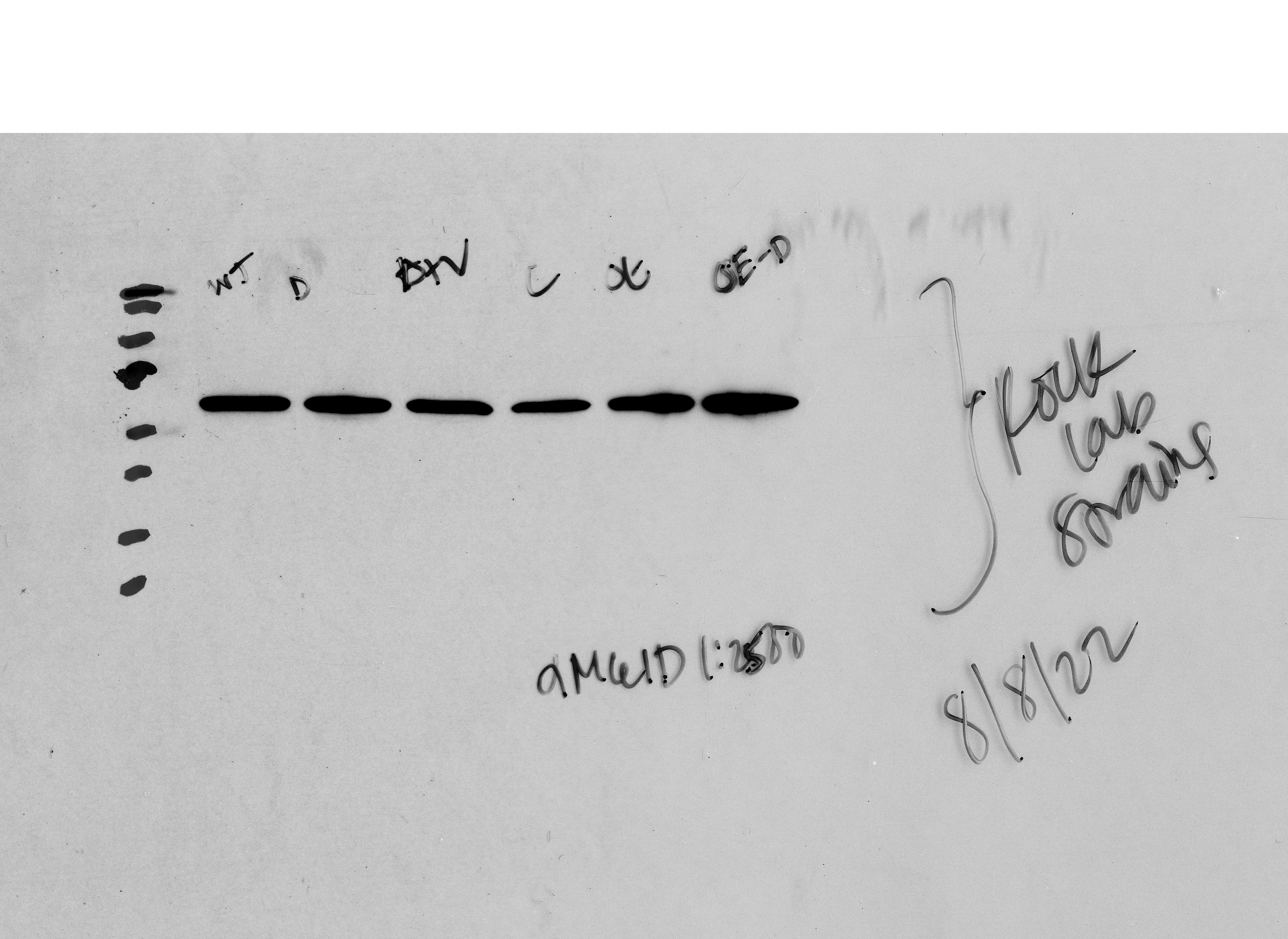

Supplement: Figure 5—figure supplement 3—source data 1. [file elife-81177-fig5-figsupp3-data1.zip › Figure 5 - figure supplement 3 - Mce1D, original.png]

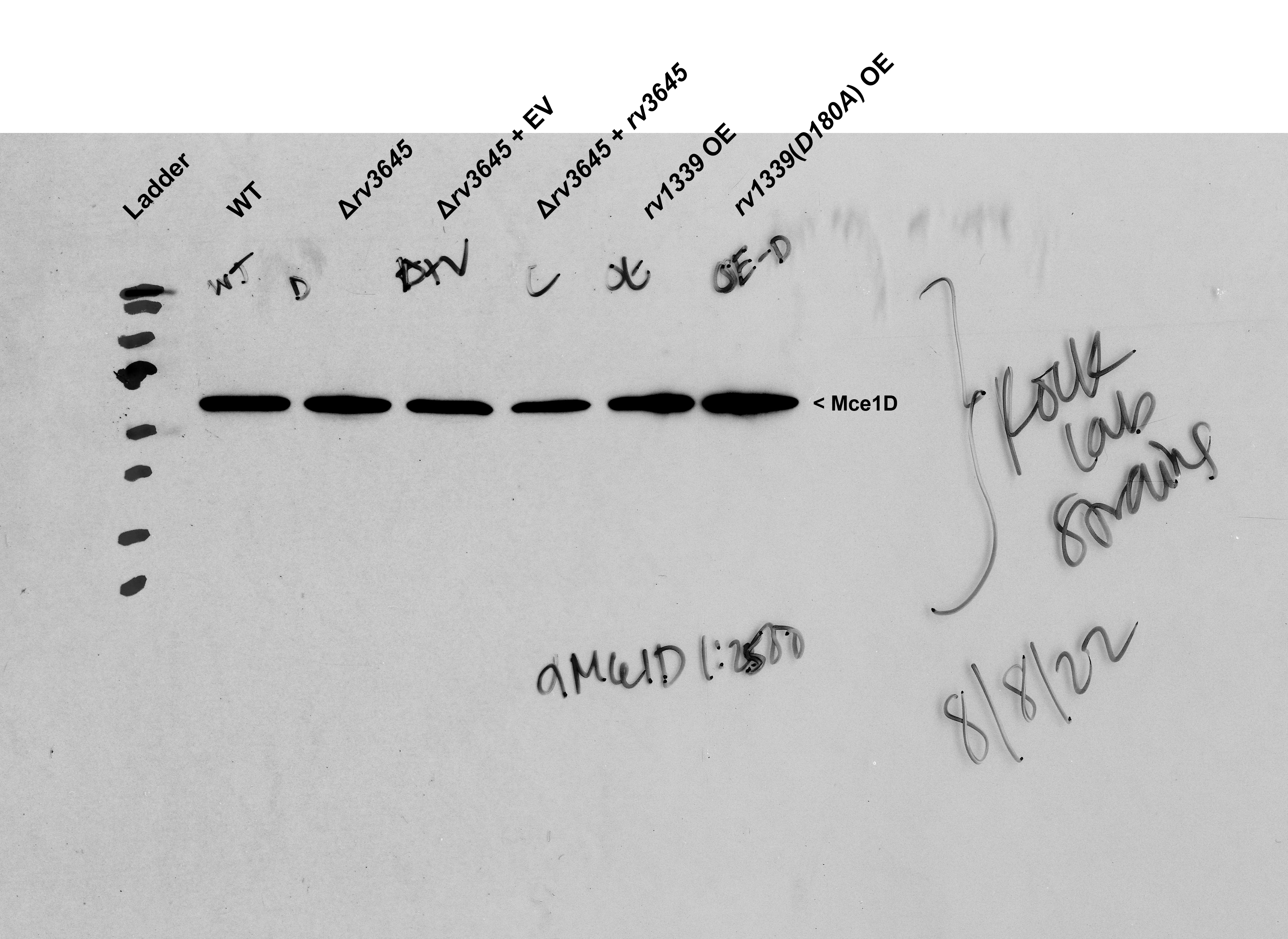

Supplement: Figure 5—figure supplement 3—source data 1. [file elife-81177-fig5-figsupp3-data1.zip › Figure 5 - figure supplement 3 - Mce1D, uncropped, labelled.png]

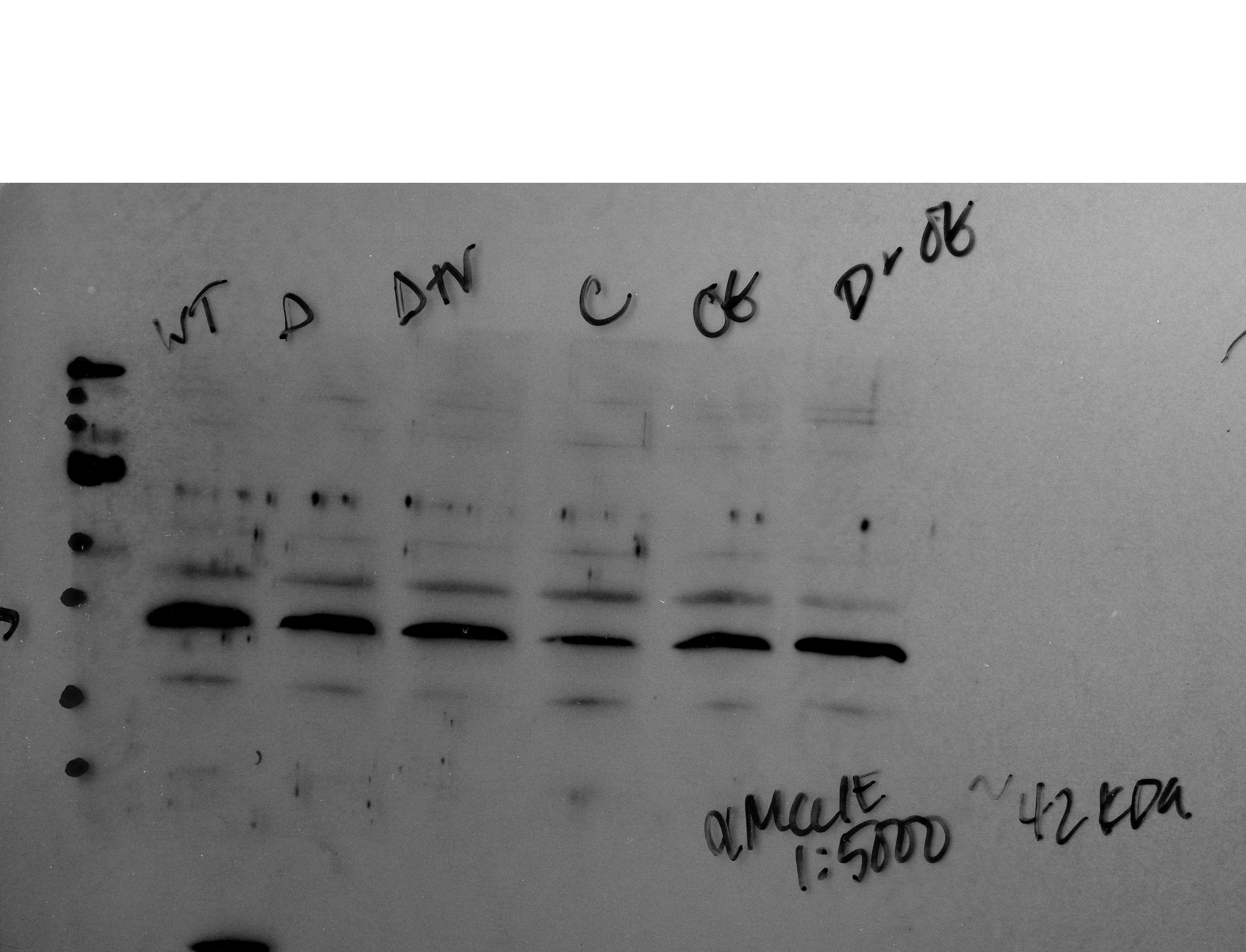

Supplement: Figure 5—figure supplement 3—source data 1. [file elife-81177-fig5-figsupp3-data1.zip › Figure 5 - figure supplement 3 - Mce1E, original.png]

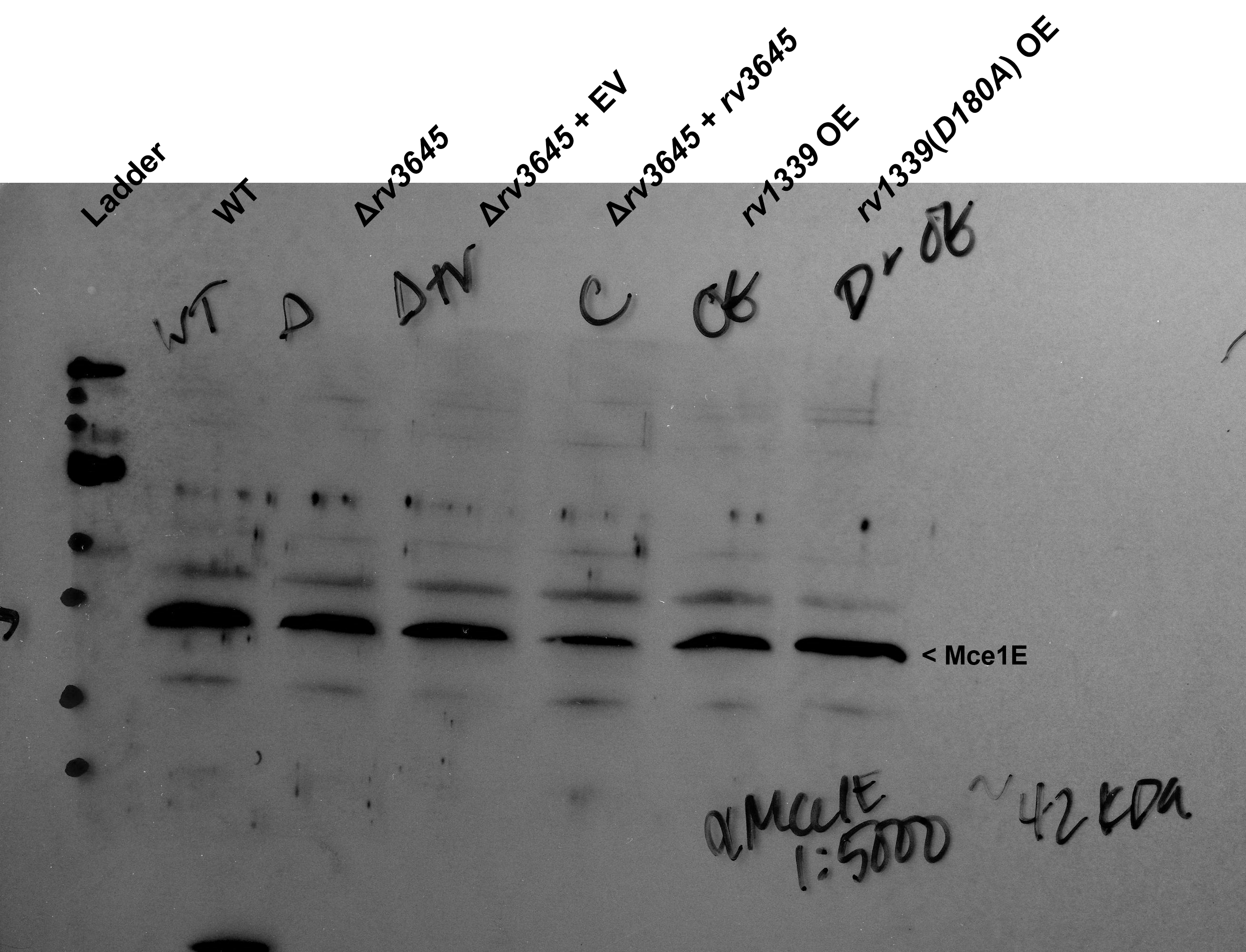

Supplement: Figure 5—figure supplement 3—source data 1. [file elife-81177-fig5-figsupp3-data1.zip › Figure 5 - figure supplement 3 - Mce1E, uncropped, labelled.png]

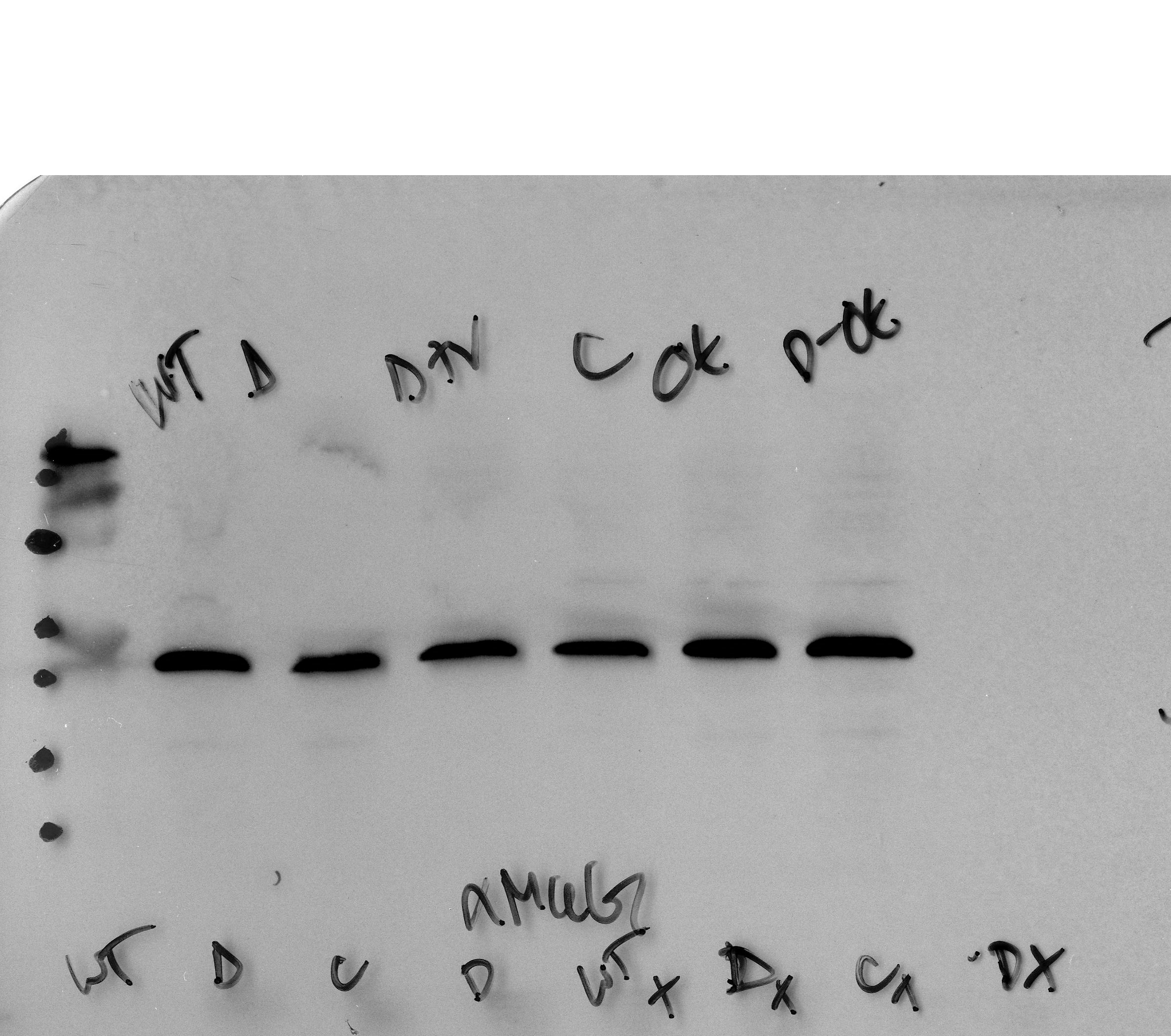

Supplement: Figure 5—figure supplement 3—source data 1. [file elife-81177-fig5-figsupp3-data1.zip › Figure 5 - figure supplement 3 - MceG, original.png]

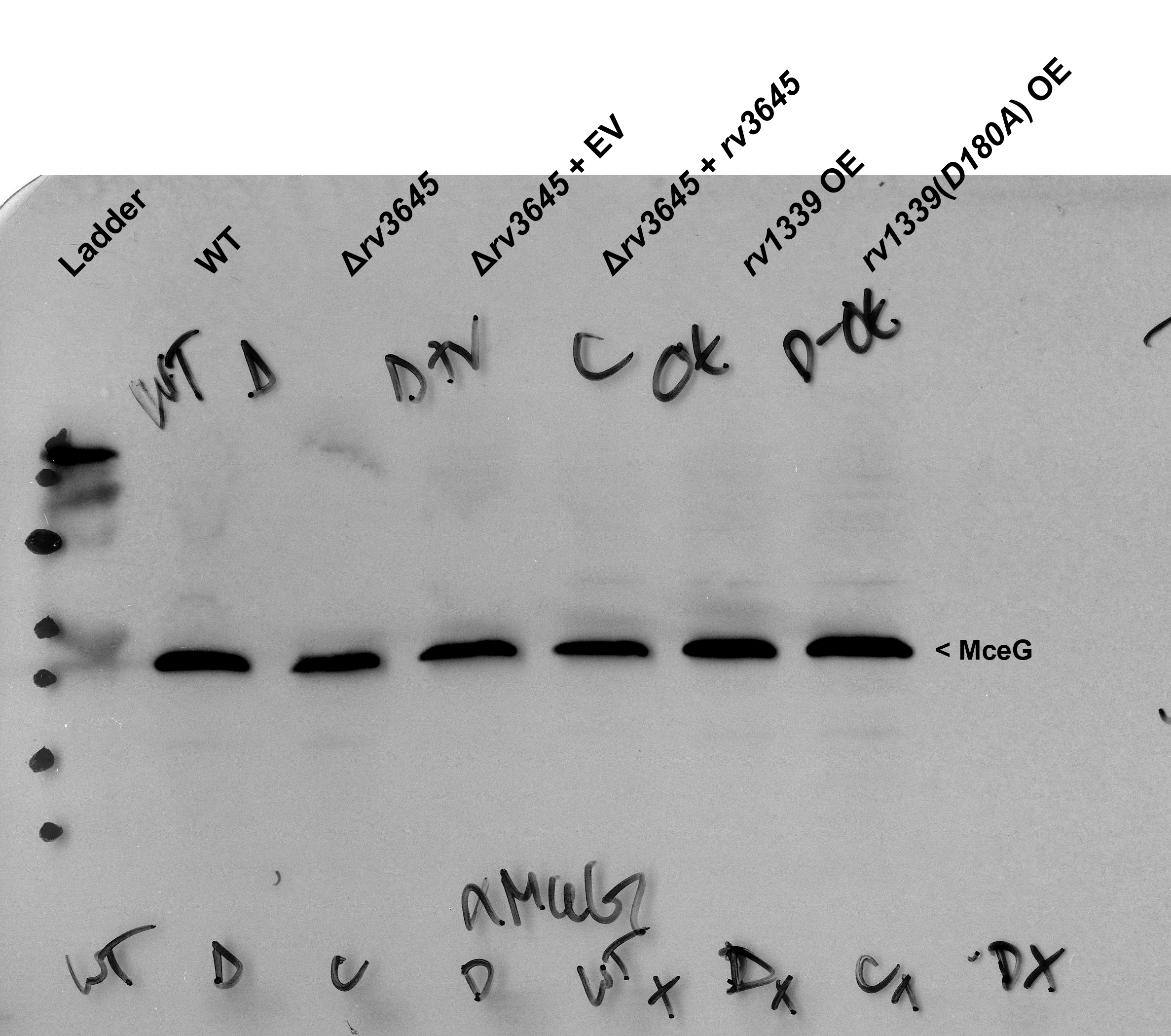

Supplement: Figure 5—figure supplement 3—source data 1. [file elife-81177-fig5-figsupp3-data1.zip › Figure 5 - figure supplement 3 - MceG, uncropped, labelled.png]
